# Design and In Vitro Study of a Dual Drug-Loaded Delivery System Produced by Electrospinning for the Treatment of Acute Injuries of the Central Nervous System

**DOI:** 10.3390/pharmaceutics13060848

**Published:** 2021-06-08

**Authors:** Luisa Stella Dolci, Rosaria Carmela Perone, Roberto Di Gesù, Mallesh Kurakula, Chiara Gualandi, Elisa Zironi, Teresa Gazzotti, Maria Teresa Tondo, Giampiero Pagliuca, Natalia Gostynska, Vito Antonio Baldassarro, Maura Cescatti, Luciana Giardino, Maria Letizia Focarete, Laura Calzà, Nadia Passerini, Maria Laura Bolognesi

**Affiliations:** 1 Department of Pharmacy and BioTechnology, Alma Mater Studiorum—University of Bologna, Via S. Donato 15, 40127 Bologna, BO, Italy; luisastella.dolci2@unibo.it (L.S.D.); rosaria.perone@libero.it (R.C.P.); 2 Interdepartmental Center for Health Sciences & Technologies (HST), CIRI-SDV, Alma Mater Studiorum—University of Bologna, Via Tolara di Sopra 41/E, 40064 Ozzano Emilia, BO, Italy; mkrakula@memphis.edu (M.K.); teresa.gazzotti@unibo.it (T.G.); marite.tondo@gmail.com (M.T.T.); giampiero.pagliuca@unibo.it (G.P.); natalia.gostynska@iit.it (N.G.); vito.baldassarro2@unibo.it (V.A.B.); luciana.giardino@unibo.it (L.G.); 3 Chemistry Department “G. Ciamician” and INST UdR of Bologna, Alma Mater Studiorum—University of Bologna, Via Selmi 2, 40126 Bologna, BO, Italy; rdigesu@fondazionerimed.com (R.D.G.); c.gualandi@unibo.it (C.G.); 4 Interdepartmental Center for Industrial Research on Advanced Applications in Mechanical Engineering and Materials Technology, CIRI-MAM, Alma Mater Studiorum—University of Bologna, Viale Risorgimento 2, 40136 Bologna, BO, Italy; 5 Department of Veterinary Medical Sciences, Alma Mater Studiorum—University of Bologna, Via Tolara di Sopra 50, 40064 Ozzano Emilia, BO, Italy; elisa.zironi@unibo.it; 6 Iret Foundation, Via Tolara di Sopra 41/E, 40064 Ozzano Emilia, BO, Italy; maura.cescatti@unibo.it

**Keywords:** multi-target drug design, ibuprofen, T3, dual-drug, nanofibers, complex diseases, TBI, SCI

## Abstract

Vascular and traumatic injuries of the central nervous system are recognized as global health priorities. A polypharmacology approach that is able to simultaneously target several injury factors by the combination of agents having synergistic effects appears to be promising. Herein, we designed a polymeric delivery system loaded with two drugs, ibuprofen (Ibu) and thyroid hormone triiodothyronine (T3) to in vitro release the suitable amount of the anti-inflammation and the remyelination drug. As a production method, electrospinning technology was used. First, Ibu-loaded micro (diameter circa 0.95–1.20 µm) and nano (diameter circa 0.70 µm) fibers were produced using poly(l-lactide) PLLA and PLGA with different lactide/glycolide ratios (50:50, 75:25, and 85:15) to select the most suitable polymer and fiber diameter. Based on the in vitro release results and in-house knowledge, PLLA nanofibers (mean diameter = 580 ± 120 nm) loaded with both Ibu and T3 were then successfully produced by a co-axial electrospinning technique. The in vitro release studies demonstrated that the final Ibu/T3 PLLA system extended the release of both drugs for 14 days, providing the target sustained release. Finally, studies in cell cultures (RAW macrophages and neural stem cell-derived oligodendrocyte precursor cells—OPCs) demonstrated the anti-inflammatory and promyelinating efficacy of the dual drug-loaded delivery platform.

## 1. Introduction

Acute lesions of the central nervous system (CNS) due to vascular and traumatic events are increasingly recognized as global health priorities because of their incidence, resulting in chronic disabilities, and consequent individual, medical, and social costs. In particular, strokes due to ischemia or hemorrhage are the second-leading cause of death worldwide, with an annual mortality of about 5.5 million. Moreover, 50% of survivors experience chronic disabilities [1]. In 2016, 27.08 million new cases of traumatic brain injuries (TBIs) and 0.93 million cases of spinal cord injuries (SCIs) have been registered worldwide, constituting a considerable proportion of the global injury burden [2]. A single brain injury can accelerate or precipitate age-related neurodegenerative diseases, including Alzheimer’s disease, Parkinson’s disease, and motor neuron disease, and repetitive mild traumatic brain injuries can provoke the development of a tauopathy and chronic traumatic encephalopathy. Furthermore, neurological, cognitive, and psychiatric sequelae are quite common, strongly impacting quality of life [3].

In both vascular and traumatic injuries, neuropathology includes a cascade of events triggered by the primary event and propagated over days and even weeks in the so-called “secondary degeneration”, which includes cellular, inflammatory, mitochondrial, neurochemical, and metabolic alterations. Secondary degeneration expands the original lesion by affecting neurons, axons, and remyelination potential, but has the potential to be reversible [3]. In spite of preclinical evidence suggesting the need for early intervention to restrict the cellular and molecular events of secondary degeneration, early therapeutic care for a stroke, TBI, or SCI does not attempt to block or limit secondary degeneration [4,5,6].

Thus, CNS injuries cannot be cured by current drug therapies.

Recently, an ever-increasing recognition of the complexity and diversity of secondary neurodegeneration has inspired therapeutic approaches directed at multiple secondary injury mechanisms [7]. In other words, considering the multifactorial nature of secondary neurodegeneration, it is unlikely that targeting a single factor will result in a significant improvement of the clinical outcome. Conversely, a polypharmacology approach that is able to simultaneously target several injury factors by the combination of drugs with complementary/synergistic effects appears to have more chance of success [8].

According to the American National Library of Medicine (NLM) terminology, polypharmacology is “the design or use of pharmaceutical agents that act on multiple targets or disease pathways”. It stands as a new and promising paradigm to develop therapies most suited to treating currently incurable complex diseases [9]. Polypharmacology is strongly supported by the emerging concepts of network medicine [10], which combines principles and approaches from systems biology and network science for understanding the causes of human diseases and finds effective treatments. Of note, polypharmacology refers to both drug combinations and so-called “multitarget” drugs [11].

One major challenge of drug combinations is to merge the pharmacokinetics and cellular uptake of the individual drug components in a way that allows precise control of the dosage and release, thereby maximizing the synergistic, combinatorial effects [12]. One strategy for overcoming this challenge is to load two drugs onto a single drug-delivery system so that they are concomitantly delivered to their sites of action [12].

On this rational basis, and building on our experience in the field, we were motivated to develop a new delivery system that would enable (i) a dual-drug delivery and (ii) a sequential drug release to effectively counteract secondary neurodegeneration. Toward this goal, we designed a polymeric delivery system based on an electrospun poly(l-lactide) (PLLA) scaffold loaded with two drugs, specifically, ibuprofen (Ibu) and thyroid hormone triiodothyronine (T3), to be implanted at the lesion site.

Here, we report the design, development, and in vitro characterization of such a polymeric delivery system, for which the in vivo proof of concept has already been obtained [13]. Particularly, we sought to design a dual-drug polymeric delivery system for Ibu and T3 that is able to release the suitable amount of the individual drugs required to produce the desired polypharmacological effect. In vitro studies in appropriate cell culture systems (RAW macrophages and neural stem cell-derived oligodendrocyte precursor—OPCs) were carried out to test the anti-inflammatory and promyelinating efficacy of the designed delivery platform. Finally, GLP-compliant studies of in vitro toxicity were also performed.

## 2. Materials and Methods

### 2.1. Materials

Poly(l-lactide) (PLLA) (RESOMER^®^ L 206 S, inherent viscosity = 0.8–1.2 dL/g), Poly(d,l-lactide-*co*-glycolide) 50:50 (PLGA 50:50) (RESOMER^®^ RG 504 H, inherent viscosity = 0.45–0.60 dL/g), PLGA 75:25 (RESOMER^®^ RG 756 S, inherent viscosity = 0.71–1.0 dL/g) and PLGA 85:15 (RESOMER^®^ RG 858 S, inherent viscosity = 1.3–1.7 dL/g) were purchased from Evonik Industries. *N,N*-Dimethylformamide (DMF), dichloromethane (DCM), and methanol (MeOH) were purchased from Sigma Aldrich. 3,3’,5-Triiodo-l-thyronine sodium salt (T3) and Ibuprofen (Ibu) were purchased from Sigma Aldrich.

For HPLC analysis, acetonitrile was of HPLC grade and was purchased from Sigma Aldrich. For LC-MS/MS analysis, methanol, acetonitrile, and formic acid were of LC-MS grade and were purchased from Sigma Aldrich (St. Louis, MO, USA). Ultrapure water was produced in-house with a Human Power I system (Seoul, Korea). Oasis^®^ HLB 200 mg SPE cartridges were purchased from Waters (Milford, MA, USA).

### 2.2. Methods

#### 2.2.1. Scaffold Fabrication

The electrospinning apparatus (Spinbow s.r.l., Bologna, Italy) comprised a high-voltage power supply, 2 glass syringes containing the polymeric solutions, each one connected to a stainless steel blunt-ended needle (inner diameter 0.51 mm), positively charged and positioned on the opposite sides of a grounded aluminum drum-type collector (diameter 5 cm) rotating at 75 rpm. The needle-to-collector distance was fixed at 20 cm. This apparatus was able to co-electrospin 2 different polymeric solutions, whose feed rates were independently controlled by 2 syringe pumps, and to collect the corresponding fibers evenly distributed on the rotating drum.

Ibu-loaded fibers were prepared by using a “single needle” electrospinning apparatus and by electrospinning the following polymeric solutions: PLGA 50:50 15% *w/v* dissolved in DCM/DMF 70:30 (*v/v*); PLGA 75:25 20% *w/v* dissolved in DCM/DMF 70:30 (*v/v*); PLGA 85:15, 8% *w/v* dissolved in DCM/DMF 70:30 (*v/v*); PLLA, 20% *w/v* dissolved in DCM/DMF 70:30 (*v/v*). Before electrospinning, all solutions were loaded with a suitable amount of Ibu to give a theoretical 5 wt%. T3-loaded PLLA fibers containing a theoretical 0.6 wt% of the drug were also produced.

The delivery system containing T3 and Ibu was produced by co-electrospinning the following two PLLA polymeric solutions: (i) PLLA 20% *w/v* dissolved in DCM/DMF 70:30 (*v/v*), with the addition of a suitable amount of Ibu to give a theoretical 5 wt% of the drug in the final fibers, and (ii) PLLA 20% *w/v* dissolved in DCM/MeOH 70:30 (*v/v*), with the addition of a suitable amount of T3 to obtain a theoretical 0.6 wt% of the drug in the final fibers. The electrospinning processing conditions used for each solution are specified in Table 1. An electrospun control sample was produced by co-electrospinning the above-described polymeric solutions not loaded with drugs. All scaffolds were produced at RT and at a relative humidity of 30% by co-electrospinning the polymeric solutions for a period of 3.5 h, giving samples with a thickness range of 170–200 μm. Samples were sterilized using γ-rays (25 KGy) prior to biological experiments.

#### 2.2.2. Morphology, Diameter Distribution, Encapsulation Efficiency, and Thermal Characterization of the Fibers

The fiber morphology was observed using a Leica Cambridge Stereoscan 360 Scanning Electron Microscope (SEM) operating at 20 kV. The samples were sputter-coated with gold prior to examination.

The fiber diameter distribution of each scaffold was determined by measuring 100 fibers, and the results are given as the average diameter ± standard deviation.

The encapsulation efficiency (EE) was calculated by the following equation:

$$EE\;\left(\%\right)=\frac{Actual\;drug\;amount}{Theoretical\;drug\;amount}\times 100$$

where the Actual drug amount was determined by the assay methods reported in Section 2.2.3 andSection 2.2.4.

Thermogravimetric analyses (TGA) were carried out using a TA Instruments (New Castle, DE, USA) Q500 Analyzer. Samples were heated at a rate of 10 °C min^−1^ from RT to 700 °C, under nitrogen flow. Differential scanning calorimetry (DSC) was carried out with a TA Instruments DSC Q2000 equipped with a refrigerated cooling system (RCS). The samples were subjected to a first heating scan at 20 °C min^−1^ from −90 °C to 200 °C, followed by quenching, and a second heating scan at 20 °C min^−1^.

#### 2.2.3. HPLC-UV Method for Ibu Determination

The amount of ibuprofen was determined by a high-performance liquid chromatography with UV detection (HPLC-UV) method with modification of previously published methods [14,15]. The HPLC system consisted of 2 mobile phase delivery pumps (LC-10ADvp, Shimadzu, Japan) and a UV-Vis detector (SPD-10Avp, Shimadzu, Japan). An autosampler (SIL-20A, Shimadzu, Japan) was used to inject samples (20 µL) onto a Kinetex 5 µm C18 100 Å (150 mm × 4.60 mm) column (Phenomenex). The HPLC system was operated at 25 °C. The optimized mobile phase comprised acetonitrile and ammonium phosphate buffer (0.02 M, pH 3, adjusted using ortho-phosphoric acid) (57:43, *v/v*). The mobile phase was filtered through a 0.22-µm Sartorius filter before use. The flow rate was 1 mL/min and the detection wavelength was set at 220 nm. The retention time of the drug resulted in 5.2 min and the chromatographic runtime was 10 min.

The determination of Ibu using the optimized method was also performed in the presence of T3. Standard solutions (PBS pH 7.4) containing a mixing of Ibu (10 µg/mL) and T3 (20 µg/mL) were analyzed. The obtained chromatograms did not present an additional peak at the Ibu retention time. Furthermore, to test the HPLC method for the determination of Ibu in culture medium, samples containing known concentrations of Ibu (1 and 10 µg/mL) in complete DMEM (Dulbecco’s Modified Eagle Medium + 10% Fetal Bovine Serum DMEM–FBS) and DMEM (without FBS) were analyzed. In both cases, the chromatograms did not show additional peaks at Ibu retention time.

The proposed method was validated according to the main requirements of the European guidelines [16] by evaluation of specificity, linearity, lower limit of quantification, accuracy, and precision (see Supplementary Materials).

#### 2.2.4. UPLC–MS/MS Method for T3 Determination

Analysis was performed on a UPLC–MS/MS system, including a Waters Acquity UPLC^®^ binary pump equipped with a built-in vacuum degasser, a thermostated autosampler, and a column heater. Chromatographic separation was achieved using a Waters Acquity UPLC^®^ BEH C18 reversed-phase column (50 × 2.1 mm, 1.7 μm) fitted with a Waters VanGuard guard column with the same packaging (Waters Corporation, Milford, MA, USA).

The mobile phase consisted of a mixture of water/methanol (90:10, *v/v*) containing 0.1% of formic acid (Solvent A) and methanol containing 0.1% of formic acid (Solvent B). The gradient (constant flow rate of 0.3 mL/min) started with 70% A and 30% B, followed by a gradient of Solvent B (2 min—75%, 2.5 min—85%, 2.6 min—95%, 2.8 min—10%, 3.5 min—75%, 4 min—60%, 5 min—30%). To wash the needle, mixtures of water/methanol (70:30, *v/v*) containing 0.1% of formic acid (weak wash) and methanol/water/acetonitrile (40:30:30, *v/v*) containing 0.2% of formic acid (strong wash) were used.

Samples were kept at room temperature in the autosampler and 10 μL was injected in “partial loop with needle overfill” mode; the column was kept at 40 °C.

The chromatographer was interfaced with a Waters Quattro Premier XE tandem mass spectrometer equipped with an ESCi multi-mode ionization source (Waters Corporation, Milford, MA, USA) and operating in positive electrospray ionization (ESI +) mode. Analysis was performed in MRM (multiple reaction monitoring) mode, following 2 transitions for T3 (the relative optimized values of cone voltage and collision energy are in brackets): 651.45 > 605.4 (33 V, 35 eV) and 651.45 > 478.7 (35 V, 30 eV).

The following parameters were applied in the tune page: the capillary voltage was set at 3.00 kV and the cone voltage at 30 V, while the source and desolvation temperatures were 130 and 450 °C, respectively. The nitrogen flow was set at 50 L/h on the cone and 500 L/h for desolvation; argon was used as the collision gas at 0.35 mL/min. Data acquisition and processing were performed using MassLynx 4.1 software (Waters Corporation, Milford, MA, USA).

The proposed method was validated according to the main requirements of the European guidelines [17] by evaluation of specificity, linearity, lower limit of quantification, accuracy, and precision (see Supplementary Materials).

### 2.3. In Vitro Drug Release Studies

Release studies were carried out on rectangular electrospun samples (about 1 × 3 cm) immersed in 10 mL of phosphate-buffered solution (PBS, 0.1 M, pH = 7.4) and incubated in an SW22 Julabo (JULABO GmbH, Lahr, Germany) shaking water bath at 37 °C for a maximum period of 15 days. At regular intervals, the PBS was completely removed and replaced with fresh buffer. Aliquots were analyzed by means of HPLC-UV and UPLC–MS/MS to determine the Ibu and T3 release, respectively. The results were reported as the cumulative release % over time for the Ibu-loaded fibers and as the Ibu and T3 cumulative release for the dual-drug loaded samples. The results represent the mean (± S.D.) of at least 3 replicates.

### 2.4. In Vitro Efficacy and In Vitro Toxicity Tests

#### 2.4.1. Cell Line Cultures (RAW 264.7 and DITNC1)

Cell lines were purchased from the American Type Culture Collection. The murine macrophage cell line RAW 264.7 (ATCC^®^ TIB-71™) was selected for efficacy studies, whereas rat astrocyte DITNC1 (ATCC^®^ CRL-2005™) was used for toxicity studies.

Cells were grown in DMEM high glucose medium (Thermo Fisher Scientific, Waltham, MA, USA) supplemented with 10% heat-inactivated fetal bovine serum (FBS—Thermo Fisher Scientific) and 1% penicillin/streptomycin (100 U mL^−1^/100 µg mL^−1^) (Thermo Fisher Scientific) at 37 °C in a humidified incubator of 5% CO_2_.

For efficacy studies, RAW 264.7 cells were seeded on a 24-well plate at a density of 12.000 cells/well and treated after 3 days in culture with conditioned medium, prepared as described below.

For GLP toxicity studies, DITNC1 cells were validated according to the doubling time and absence of mycoplasma contamination. Soluble molecules and conditioned medium for toxicity studies were used as described in the dedicated sections.

#### 2.4.2. Cell Cultures of Primary Neural Stem Cell-Derived OPCs

All animal protocols described herein were carried out according to the European Community Council Directives (86/609/EEC) and comply with the guidelines published in the NIH Guide for the Care and Use of Laboratory Animals.

Fetal neural stem cells (NSCs) were isolated from E.13.5 rat forebrain, as already described, with some modifications [18]. The tissues were incubated in non-enzymatic dissociation buffer (Sigma-Aldrich, Saint Louis, MO, USA) at 37 °C for 15 min, and mechanically dissociated cells were resuspended in serum-free medium (DMEM/F12 GlutaMAX 1×; 8 mmol/L HEPES; 100 U/100 μg penicillin/streptomycin; 0.1 × B27; 1 × N-2; 20 ng/mL bFGF; 20 ng/mL EGF; Thermo Fisher Scientific) and plated in suspension at a density of 10 cells/µL, in flasks (Nunc, Roskilde, DK) kept vertical to avoid cell adhesion until the neurospheres reached an average diameter of about 100 µm.

The primary neurospheres were centrifuged at 300× *g* for 5 min and the pellet was mechanically dissociated by pipetting. The cells were counted and plated again at a density of 10 cells/µL in OPC medium (DMEM/F12 GlutaMAX 1×; 8 mmol/L HEPES; 100 U/100 μg penicillin/streptomycin; 0.1 × B27; 1 × N-2; 20 ng/mL bFGF; 20 ng/mL PDGF; Thermo Fisher Scientific) to obtain oligospheres. When the spheres reached a diameter of 100 µm, they were mechanically dissociated and single cells were plated at a density of 3000 cells/cm^2^ on poly-d,l-ornithine (50 µg/mL)/laminin (5 µg/mL; Sigma-Aldrich) coating in OPC medium.

In the control groups, to induce oligodendrocyte differentiation and maturation, the standard assay was used, replacing the OPC medium with the oligodendrocyte differentiation medium (DMEM/F12 GlutaMAX 1×; 8 mmol/L HEPES; 100 U/100 μg penicillin/streptomycin; 0.1 × B27; 1 × N-2; 50 nM T3; 10 ng/mL CNTF; 1× N-acetyl-l-cysteine—NAC; Thermo Fisher Scientific) following 3 DIVs. To test the efficacy of the PLLA electrospun scaffolds loaded with T3 and ibuprofen to induce OPC differentiation, constant surfaces of vehicle-PLLA and ibuprofen-T3-PLLA electrospun scaffolds were submerged in the wells using a 24 well plate ring, without touching the bottom of the well-containing cells. OPCs were exposed for 12 DIVs and then immunocytochemistry was performed to quantify the differentiation induced by the different culture conditions.

#### 2.4.3. Immunocytochemistry

Specific antibodies were used to perform a qualitative morphological analysis of RAW 264.7 cells (b-actin) exposed to LPS and for the quantitative analysis of NSC-derived OPC differentiation (CNPase and MBP, mature oligodendrocytes markers). The cells were fixed with 4% for 20 min at room temperature and then incubated with the blocking solution (PBS, triton 0.3%, BSA 1%, donkey normal serum 1%). The cells were then incubated overnight at 4 °C with primary antibodies (Anti b-actin, goat, Santa Cruz, 1:200; Anti-CNPase, mouse, Millipore, 1:250; Anti-MBP, rabbit, Dako, 1:250) diluted in PBS triton 0.3%. For b-actin staining, fixation was followed by 5 min of incubation in cold methanol, as indicated by the manufacturer. After 3 washes with PBS, coverslips were incubated with secondary antibodies (Anti-mouse IgG RRX-conjugated, Jackson Immunoresearch; Anti-rabbit IgG Alexa Fluor 488-conjugated, Molecular Probes; anti-goat IgG DyLight 488-conjugated, Thermo Fisher Scientific) at 37 °C for 2 h. The cells were also incubated with the nuclear dye Hoechst 33,258 (1 μg/mL) to identify the nuclei. Coverslips stained with only the secondary antibody solutions were used as controls.

Fluorescence microscopy observations and photography were performed using a Nikon Eclipse E600 microscope equipped with the digital CCD camera Q Imaging Retiga-2000 RV (Q Imaging, Surrey, BC, CA) and Nis-Elements AR 3.2 software. For the OPC differentiation analysis, for each coverslip, 5 random pictures were acquired and the percentages of CNPase and MBP-positive cells were calculated on the total cells (Hoechst-stained nuclei) in each field.

#### 2.4.4. Preparation of Conditioned Medium for Efficacy Studies on RAW 264.7

Sterilized samples (1.5 cm × 0.5 cm) of the scaffolds loaded with both Ibu and T3 (PLLA + Ibu + T3) and blank scaffolds (PLLA) were immersed in 1 mL of complete growth medium for 3 days at 37 °C with shaking (50 rpm). Media with Ibu at a final concentration of 200 μM and T3 at 250 nM with blank PLLA electrospun scaffolds were incubated under the same conditions and served as control samples. At the end of the incubation, the cells were treated with conditioned medium and lipopolysaccharide (LPS, 500 ng/mL) for 24 h. From each group, 3 samples were analyzed.

#### 2.4.5. GLP Toxicity Assay for Ibuprofen and T3 Combination

DITNC1 cells (rat astrocytes, type 1 phenotype) were seeded in 4 different 96-well plates (Thermo Fisher Scientific) at a density of 10,000 cells/well. Each plate was set up based on the GLP guidelines for Good Cell Culture Practice; all the external perimeter wells contained only water to reduce the evaporation of the culture medium, groups of treatments (8 different concentrations) were set up in columns (6 replicates per group) and the first and the last columns corresponded to the vehicle-treated groups to check for the variability in the same plate. A plate treated with sodium dodecyl sulfate (SDS) was used as a positive control plate for cytotoxicity (test concentrations: 0.08, 0.12, 0.17, 0.25, 0.37, 0.56, 0.80, and 1.18 mM). A dose-response curve plate for T3 was included in the study, taking into account that the concentration used for the OPC differentiation assay was 50 nM (test concentrations: 15.7, 23.1, 34.0, 50.0, 73.5, 108.0, 158.8, and 233.4 nM). To test both the ibuprofen toxicity and if it was influenced by the presence of T3, ibuprofen was tested alone or in combination with the fixed 50 nM dose of T3 (ibuprofen test concentrations: 0.26, 0.39, 0.58, 0.86, 1.26, 1.85, 2.72, and 4.00 mM).

After 24 h following the treatments, the MTT assay was carried out to quantify the cell viability. The culture medium was removed from each well, 100 µL of MTT solution (0.5 mg/mL in OptiMEM) was added, and the cells were incubated in standard conditions (37 °C, 5% CO_2_). After 3 h, 100 µL of solubilization solution (isopropanol 80%, HCl 1M 10%, Triton 10%) was added to each well and the plates were incubated for 1 h under shaking. Absorbance was detected by a plate reader (Biorad) at 570 nm. Wells containing only medium were used to measure the background value.

For the quality control, the average values of 2 columns of the vehicle-treated groups for each plate were compared (variability < 15%).

To calculate the percentage of cell viability for each plate, the background value was subtracted from the absorbance value of all the wells. The averages of the 2 columns of the vehicle-treated groups were used as a control (100%) and all the values for each well were calculated as a percentage of the control.

The values for each dose-response curve have been used as inputs by using GraphPad Prism software (v.7), as in the non-linear regression (Sigmoidal, 4PL, X is log(concentration)), to calculate the IC_50_ value.

#### 2.4.6. GLP Toxicity Assay for Ibuprofen and T3 Released from PLLA Electrospun Scaffolds

The objective of this experiment was to test the toxicity of the conditioned medium obtained from PLLA electrospun scaffolds loaded with ibuprofen and T3. To perform a dose-response curve, different conditioning times were used; a constant surface of the electrospun scaffold (4 cm^2^) was submerged in 1 mL of culture medium incubated in standard culture conditions (37 °C, 5% CO_2_). For this experiment, cells were seeded in 24-well plates (20,000 cells/well) and different times of exposure were used to perform a dose-response curve (1, 2, 3, 4, 5, 6, 7, and 8 days). For the vehicle-treated group, a culture medium was conditioned using PLLA, and for a control group, a not-conditioned medium, incubated for 8 days in standard conditions, was used. For each group, 3 replicates were performed.

The cells were exposed for 24 h to the conditioned medium, and the MTT assay was performed as described above, using 250 µL of solutions per well. The volume from each well was moved in 2 96-well plates for the measure of the absorbance.

A 96-well plate treated with SDS was included in the experiment, as described above, as a positive cytotoxicity control assay.

Quality controls and viability calculations were performed as described above, using as a normalization group both the vehicle and the non-conditioned treated groups.

### 2.5. RNA Isolation, Reverse Transcription RT-PCR, and Real-Time PCR

Total RNA isolation was performed by using the RNeasy Micro kit (Qiagen, Milan, Italy) following the manufacturer’s instructions. First-strand cDNAs were obtained using the iScript™cDNA Synthesis Kit (BioRad). An RNA sample with no reverse transcriptase enzyme in the reaction mix was processed as a no-reverse transcription control sample. Semi-quantitative real-time PCR was performed using the CFX96real-time PCR system (BioRad, CA, USA).

### 2.6. Cytokine Assay

Tumor necrosis factor-α (TNF-α) was quantified in RAW 264.7 cell culture surnatants, using xMAP technology and a Bio-Plex Pro™ Cytokine-plex Assay (Bio-Rad; Milano, Italy) kit. Briefly, after treatments, the cellular surnatants were collected and centrifuged at 4000× *g* for 10 min at 4 °C before being processed. Then, 50 μL were incubated with a specific monoclonal antibody-conjugated bead population for 30 min at RT, washed beads were incubated with detection antibody solution at RT for 30 min, then with the streptavidin–phycoerythrin-conjugated solution (RT, 10 min). After washing, the beads were resuspended in the assay buffer, shaken for 1 min, and then a reading was performed on the MAGPIX instrument. The results were analyzed with xPONENT 4.2^®^ software and expressed as pg/mL.

## 3. Results and Discussion

### 3.1. Design of the Dual Drug-Loaded Delivery System

Detailed analysis of the problem led us to formulate the hypothesis that coadministration of Ibu and T3 at the lesion site by a local delivery platform may simultaneously contrast neuroinflammation and induce remyelination via oligodendrocyte maturation, with enhanced therapeutic efficacy.

It is well established that neuroinflammation is a key pathological response to brain injury, as well as an important manipulatable aspect of secondary degeneration [19]. Moreover, tissue inflammation has been demonstrated to block the terminal differentiation of oligodendrocyte precursor cells (OPCs), the cells responsible for myelin repair [16,20]. However, the use of systemic anti-inflammatory drugs has not proved efficacious for either TBI or SCI [21]. The key to developing efficient treatments is to minimize the detrimental effects of neuroinflammation while promoting the beneficial ones, and at the same time, to create optimal conditions for regeneration and repair after injury [21]. In this respect, Ibu shows strong anti-inflammatory activity by inhibiting IL-1β, IL-6, IL-10, and prostaglandins. However, there were mixed results concerning edema and the improvement of brain function in TBI models [21]. Notably, Ibu belongs to the subset of COX-inhibitors able to inhibit Rho-A. Rho-A inhibition is a promising therapeutic target in SCI because it has been shown to increase the myelination of axons and promote axonal elongation and sprouting. However, the inactivation of Rho-A must occur acutely after injury and a delayed administration does not show any regenerative effect [22]. T3 enhances remyelination in chronic demyelinating inflammatory disease and in brain injuries. This concept is consolidated in the literature, confirmed in several models of demyelination disorders, such as the animal models for multiple sclerosis, brain and spinal cord traumatic injuries, vascular lesions in infants and adults, viral demyelination, and so forth. [23,24,25]. It has also led to a phase I clinical trial for multiple sclerosis [26]. On this basis, we were motivated to develop a dual-drug delivery system for CNS lesion application based on nanofibers with the ability to carry Ibu and T3 as anti-inflammation and remyelination drugs.

Specifically, the delivery system was produced using electrospinning technology. Electrospinning is an increasingly popular technology that uses electrostatic forces to produce continuous nanometric and micrometric fibers, typically assembled into nonwoven mats [27]. The great interest in this technology arises from the simplicity of the set-up, the cost-effectiveness of the apparatus, and the versatility of the process that allows for producing materials that possess a wide range of chemical–physical and mechanical properties. Moreover, multicomponent fibers or drug-loaded and particle-loaded fibers as well as “composite” non-woven meshes can be easily obtained by concomitantly electrospinning different materials. Another advantage of electrospinning is the possibility to tune micro/nano-architecture—in terms of fiber dimension, surface porosity, and orientation—by tuning the process parameters. Electrospun nanofibrous assemblies show unique features, such as high specific surface area, high porosity, and similarity to the extracellular matrix structure. Thanks to these characteristics, they find useful applications in a variety of fields, including tissue engineering and drug release [28,29].

As a material for the preparation of the fibers, we selected PLLA and PLGA copolymers at different lactide/glycolide ratios. These aliphatic polyesters are widely utilized for the production of drug-loaded electrospun fibers due to their excellent biocompatibility, biodegradability, and non-toxic properties [30]. Moreover, they are approved by the FDA and EMA for parenteral administration. Furthermore, the drug release rate from PLLA and PLGA systems can be modified by varying the polymer properties (lactide/glycolide ratio, molecular weight, and crystallinity) [31].

The choice of a local delivery system to be implanted at the lesion site immediately after lesion occurrence was also motivated by the following considerations: (i) T3 does not distribute preferentially to the CNS from a systemically administered dose. As such, it is difficult to separate CNS from peripheral hormone effects, with the lack of a therapeutic window separating the desired therapeutic CNS actions from the risk of hyperthyroidism after systemic delivery; (ii) similarly, Ibu is not routinely used for brain injuries due to a poor penetrance across the blood–brain barrier into the cerebrospinal fluid (CSF), and potential complications including gastric ulceration and increased bleeding risk [32].

To rationally design the delivery system, preliminary studies on cell culture were instrumental in identifying the molar concentration and ratio of the two drugs in combination that are necessary to achieve the desired effect without toxicity. Regarding the dose of T3 necessary for OPC differentiation induction, 50 nM of soluble T3 is the dosage proven to efficiently activate the OPC differentiation mechanisms [18,20,33]. Conversely, we purposely ran in vitro tests using the murine macrophage cell line RAW 264.7 exposed to LPS to determine the Ibu dose necessary for the anti-inflammatory effect [34].

We also performed a toxicity analysis of the combination of the two drugs on the DITNC1 astrocyte cell line, according to the GLP guidelines.

**Figure 1:**
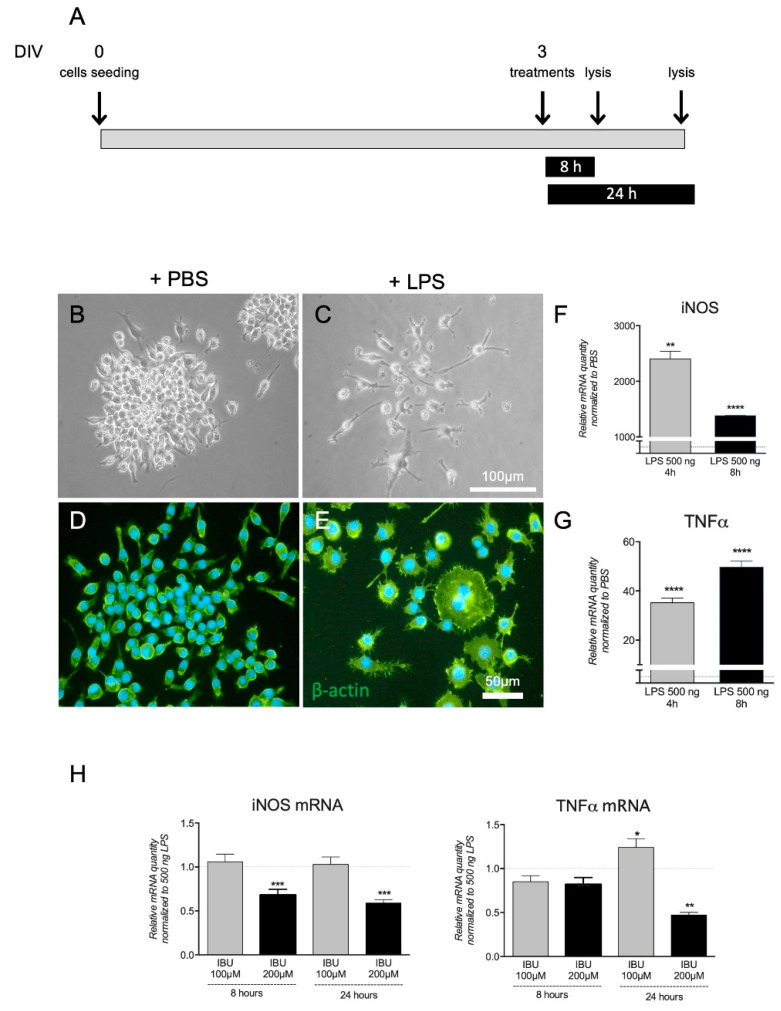
Effect of Ibu exposure on LPS-induced molecular regulations in macrophages. (**A**). Schematic representation of the experiment timeline. Morphological evaluation of RAW 264.7 cells exposed to PBS (**B**,**D**) or LPS (**C**,**E**). Image includes pictures in bright field (**B**,**C**) and in epifluorescence microscopy by b-actin immunostaining (**D**,**E**). The graphs report the expression level of iNOS (**F**) and TNF-α (**G**) mRNA, expressed as a variation (fold of increase) with respect to PBS-treated culture, indicated by the white horizontal bar. The graphs report the effect of soluble Ibu at two different concentrations and two different post-exposure times on iNOS and TNF-α mRNA expression (**H**). Statistical analysis: Student’s *t*-test, * *p* < 0.05, ** *p* < 0.01, *** *p* < 0.001, **** *p* < 0.0001.

To determine the concentration of Ibu to be loaded on the electrospun scaffold, macrophages exposed to an inflammatory challenge were used. First, the cells after LPS (500 ng) exposure were characterized, and then concentration-dependent and time-course experiments with Ibu in solution were performed (Figure 1A).

Results of the LPS stimulation are reported in Figure 1B–G, where the morphology of the cells exposed to the control conditions (PBS; B) and LPS (C) in the bright field, as well as visualized by β-actin immunostaining (D and E, respectively), are shown. The cell cultures exposed to LPS displayed the typical cluster disaggregation, and single cells enlarged, retracted elongations, and assumed a phagocytic morphology. Regarding the pro-inflammatory markers evaluation, iNOS and TNF-α mRNA expression levels dramatically increased already 4 h after exposures, remaining up-regulated over the observational time (Figure 1F,G).

We then investigated if soluble Ibu was able to counteract the LPS-induced gene expression regulation at two concentrations (100 and 200 µM) and two time-points (8 and 24 h). The highest Ibu concentration (200 µM) and the longest treatment time (24 h) resulted in significant down-regulation of both iNOS and TNF-α mRNA levels (Figure 1H), and these doses were chosen for further experiments.

Since GLP-certified cytotoxicity studies are mandatory for new combinations of approved drugs [35], we performed a first GLP study to test the combinatorial treatment of Ibu and T3. This was to assess the in vitro safety of the drugs, designed using scalar doses of Ibu and a fixed dose of T3. Astrocytes were chosen as the cellular test system (DITNC1 cell line, rat astrocytes), being CNS cells activated by inflammation, responsible for the reactive gliosis during the acute phase of the lesion [36] and for scar formation during the late phase of the lesion [37]. Astrocytes are also the key character in controlling the level of active TH in the CNS [38].

**Figure 2:**
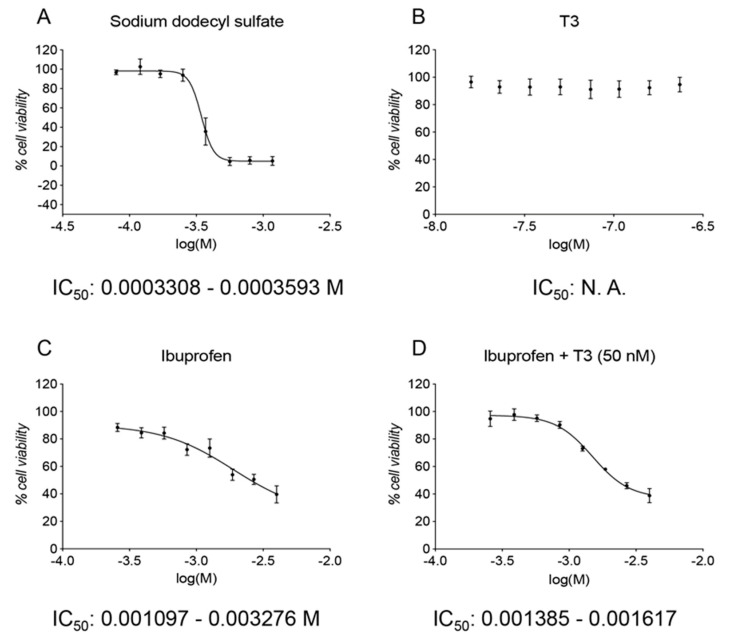
GLP toxicity study of the Ibu–T3 combination. The graphs show the cell viability normalized on the vehicle control group (100%) of cells exposed to different doses of sodium dodecyl sulfate (**A**), triiodothyronine (**B**), ibuprofen (**C**), and ibuprofen in the presence of a fixed dose of triiodothyronine (**D**). Concentrations on the x-axis are shown as the log of the Molarity; log(M). For each graph, the IC_50_ value is shown as Molarity (**A**,**C**,**D**) or as N. A. (not-applicable; (**B**)) if it was not possible to calculate the value.

The cell system resulted in sensitivity to cell death induction by the reference substance (SDS), showing an IC_50_ of 0.0003448 M (0.0003308–0.0003593 M) (Figure 2A). T3 at all the tested concentrations showed no cytotoxic effect (Figure 2B). Ibuprofen treatments never reached the 0% of cell viability, however, the IC_50_ of the plate treated with Ibu alone resulted in 0.001896 M (0.001097–0.003276 M) and, when in combination with T3 50 nM, remained constant, showing an IC_50_ of 0.001497 M (0.001385–0.001617 M) (Figure 2C,D).

### 3.2. Development and Characterization of the Ibu-Loaded Electrospun Fibers

Drug release from electrospun polymer scaffolds is strongly influenced by scaffold proprieties. Both the chemical–physical properties of the material and the morphology of the construct, in terms of fiber size, alignment, as well as porosity, play an important role in the final drug release kinetics. Thus, with the aim of selecting the most suitable drug-loaded electrospun polymeric scaffold, in the first part of the work, PLLA homopolymers and several PLGA copolymers with different lactide/glycolide ratios were investigated. Scaffolds were fabricated using the electrospinning parameters reported in Table 1, which were carefully optimized based on the properties of each polymeric solution to achieve a stable and reproducible electrospinning process, and to obtain homogeneous and regular fibers. All tested PLGA polymers have a glass transition temperature (Tg) in the range of 40–60 °C, depending on the copolymer composition, and they are completely amorphous, even at high molar concentrations of lactide, being normally synthetized using a racemic mixture of the D-PLA and L-PLA enantiomers. Similarly, PLLA, despite being isotactic, is completely amorphous when processed by electrospinning, as previously described [39,40].

In order to investigate the effect of fiber diameter on drug release kinetics, PLLA fibers with nanometric and micrometric diameters were produced by increasing the flow rate parameter of the electrospinning process (Table 1).

The majority of the fibers had an actual Ibu content quite close to the theoretical one, and therefore, the EE% was around 100% for almost all the samples (Table 1).

**Figure 3:**
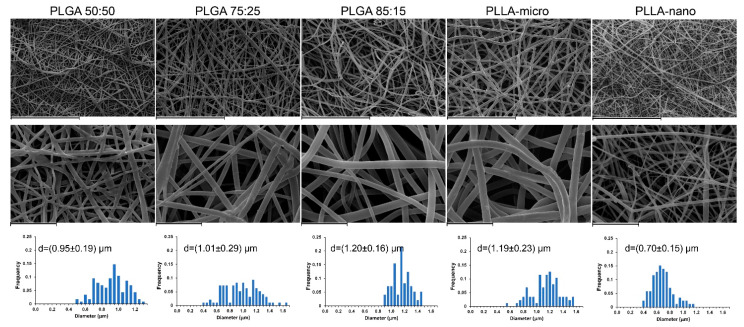
SEM images of the different electrospun scaffolds loaded with 5 wt% of Ibu at different magnifications and the corresponding fiber diameter distributions. Scale bars: 60 µm (first raw) and 10 µm (second raw).

Figure 3 shows SEM images of the Ibu-loaded scaffolds together with the corresponding fiber diameter distributions. The fibers were randomly oriented and showed continuous, smooth, and bead-free morphology, confirming the proper choice of the processing parameters. All types of PLGA fibers and PLLA-micro fibers have micrometric diameters (PLGA 50:50: 0.95 ± 0.19 µm; PLGA 75:25: 1.01 ± 0.29 µm; PLGA 85:15: 1.20 ± 0.16 µm, PLLA-micro 1.19 ± 0.23 µm), whereas PLLA-nano sample is made by submicrometric diameters (0.70 ± 0.15 µm).

**Figure 4:**
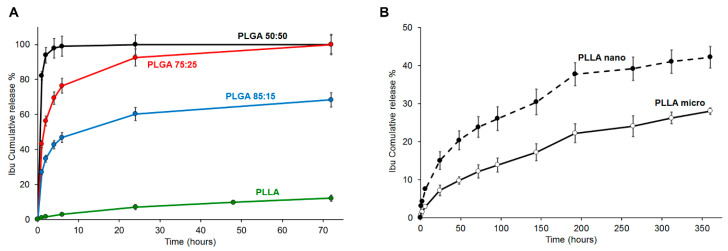
(**A**) Ibu release from scaffolds of different materials with similar fiber diameters: PLGA 50:50 (black), PLGA 75:25 (red), PLGA 85:15 (blue), and PLLA (green); (**B**) Ibu release from PLLA fibers with either micrometric (solid line) or nanometric (dot line) diameters.

Figure 4A compares the cumulative release of Ibu from the different polymeric scaffolds. It is evident that the polymer composition had a pronounced effect on the Ibu release from the fibers. PLGA fibers released around 80% of the drug in 24 h. As expected, the drug release rate increased with the increase of glycolide content in the copolymer, due to the increase of the hydrophilic character of the polymer. However, all PLGA fibers showed a burst release, whose extent was again correlated to the matrix hydrophilicity (after 1 h, the Ibu release was about 82%, 43%, and 27% from PLGA 50:50, PLGA 75:25, and PLGA 85:15, respectively). The PLLA system exhibited better performances, with an Ibu release <5% after 1 h, followed by a controlled release phase.

In addition to polymer composition, fiber morphology (and specifically, fiber diameter) is another property that may be varied in order to control drug release kinetics. Then, to evaluate the effect of the fiber size, the drug release of nanometric and micrometric fibers based on PLLA and containing a theoretical 5% Ibu was compared. Both materials (micro and nano) showed similar release profiles (Figure 4B). The decrease of PLLA fibers diameters, and the consequent increase of surface-to-volume ratios, determined an increased Ibu release (Figure 4B); the cumulative drug release after 14 days was 42% and 28% for PLLA-nano and PLLA-micro, respectively.

### 3.3. Preparation and Characterization of the Dual Drug-Loaded Delivery System

Based on the in vitro Ibu release results, the PLLA nanofiber system, having a theoretical 5% Ibu loading, was selected for the production of electrospun fibers containing both Ibu and T3, also taking the lower water solubility of T3 compared to Ibu into account (3.96 mg/L vs. 21 mg/L) [41]. Thermal characterization was carried out to verify that the loading of either Ibu or T3, as well as the sterilization treatment, did not remarkably affect the physical properties of the PLLA fibers (see Supplementary Materials, Figure S3).

**Figure 5:**
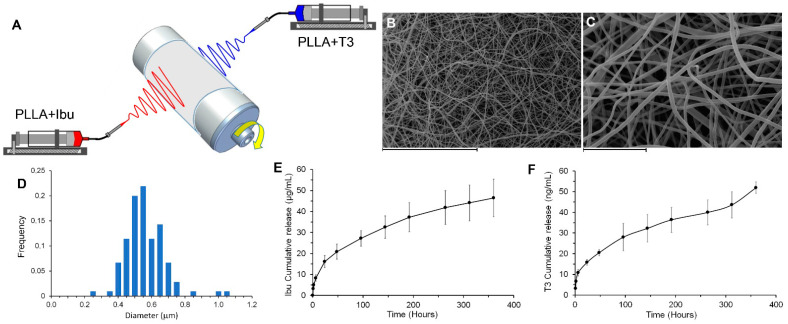
(**A**) Scheme of co-electrospinning instrumental set-up for the production of the dual-drug delivery electrospun system consisting of a fibrous scaffold composed of PLLA fibers loaded with Ibu at 5 wt% and of PLLA fibers loaded with T3 at 0.6 wt%. (**B**,**C**) SEM images of the delivery system at different magnifications (Scale bars: 60 µm (**B**) and 10 µm (**C**)); (**D**) fiber diameter distribution. (**E**) Ibu cumulative release in PBS over 400 h; (**F**) T3 cumulative release in PBS over 400 h.

To prepare the dual-drug delivery electrospun system, the drugs were individually loaded by “direct blending” in different polymeric solutions that were simultaneously co-electrospun on the same collector to gain a fibrous scaffold composed of differently loaded fibers (Figure 5A).

The actual content of the two drugs loaded in the nanofibers was inferior to the theoretical one (Ibu 3.4% vs. 5.0% and T3 0.2% vs. 0.6%). This result can be explained considering the higher variability of the two-nozzle process compared to the single-nozzle one. The interaction between jets carrying the same charge causes the jets to repel each other, resulting in lower process control and uneven fiber deposition. However, repulsion between jets could be reduced through proper design of the spinneret, by increasing the distance between the nozzles or making use of auxiliary electrodes to concentrate the electric field.

Figure 5B,C shows SEM images of the final dual drug-loaded delivery system consisting of PLLA + Ibu and PLLA + T3 fibers with nanometric diameters (mean diameter: 580 ± 120 nm, Figure 5D).

Panels E and F of Figure 5 show the cumulative release of Ibu and T3 from the composite scaffold, showing that 48 µg/mL of Ibu were progressively released over 14 days, with an estimated daily release of 3.4 µg/mL, and that 50 ng/mL of T3 was released over the same time interval, with an estimated daily release of 3.5 ng/mL. In these experiments, a surface of 3 cm^2^ was immersed in 10 mL of PBS (at 37 °C with shaking).

In order to better approach the cell culture experiments, release studies performed in PBS (Figure 5E,F) were repeated using a condition comparable to the in vitro studies. Briefly, the conditioned medium was prepared by dipping the Ibu–T3-loaded electrospun scaffold in culture medium up to 14 days, and drug concentrations at 2, 7, and 14 days were measured. After 2 days, the Ibu concentration was 34 µg/mL, corresponding to 165 µM, and thus, we prolonged the conditioning time to 3 days to get closer to the reference concentration of 200 µM. After 7 and 14 days, the Ibu concentration was 61.0 µg/mL (corresponding to 296 µM) and 52.0 µg/mL (corresponding to 252 µM), respectively. Concerning T3, and considering that the OPC differentiation protocol requires 12 days of T3 exposure, the release at 7 and 14 days confirmed an appropriate concentration (51 nM). Thus, these results combined with the results presented in Figure 1 demonstrate that the final Ibu–T3-loaded PLLA nanofibers provide the in vitro target sustained release. According to the available data, the expected CNS tissue concentration of Ibu after systemic administration of 60 mg/kg in constant infusion should be ~0.7 µg/g in rat [42], while the cerebrospinal fluid concentration in human is ~0.5 µg/mL after a single 800 mg oral administration [43]. The physiological T3 concentration in the CNS should be ~15 ng × g^−1^ CNS tissue [24,44].

### 3.4. In Vitro Efficacy and In Vitro Toxicity Tests of the Dual Drug-Loaded Delivery System

After having confirmed that the electrospun scaffold contains and releases the target combination of Ibu and T3, we next examined its efficacy by using the same in vitro system set up for determining the Ibu concentration.

**Figure 6:**
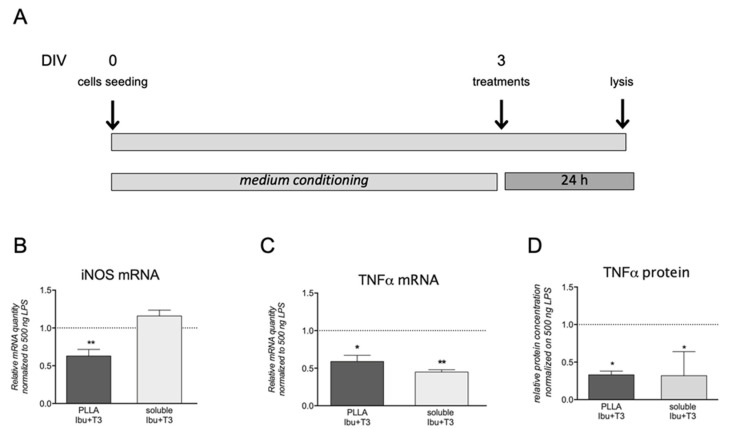
Effect of the PLLA+Ibu+T3 conditioned medium on LPS-induced molecular regulations in macrophages. (**A**) Schematic representation of the experiment timeline. (**B**–**D**) The graphs show the effect of ibuprofen and T3 derived by PLLA + IBU + T3-conditioned medium on the mRNA expression of iNOS (**B**) and TNF-α (**C**) genes, and TNF-α protein (**D**). The graphs report the expression levels expressed as variations (fold of change) with respect to LPS-PLLA exposed culture, indicated by the horizontal bar. Statistical analysis: Student’s *t*-test, * *p* < 0.05, ** *p* < 0.01.

For this experiment, the conditioned culture media containing Ibu and T3 released from the PLLA scaffolds were prepared following the ISO 10993-12:2012 guidelines (Figure 6A). The treatments using the conditioned medium were demonstrated to be effective in counteracting LPS-induced up-regulation of iNOS and TNF-α mRNA expression level (3 DIV), with similar (TNF-α) or even higher (iNOS) efficacy of the drugs applied in the solution (Figure 6B,C). Additionally, PLLA + Ibu + T3-conditioned medium significantly reduced the TNF-α protein release with respect to PLLA alone, with the same efficacy of the two drugs applied in solution (Figure 6D).

**Figure 7:**
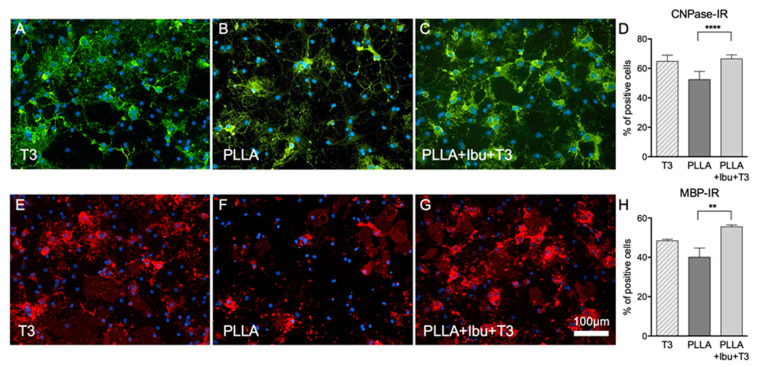
Effect of PLLA–Ibu–T3-conditioned medium on OPC maturation. Immunostaining of CNPase after exposure to soluble T3 (50 nM) (**A**), PLLA alone (**B**), PLLA + IBU + T3 (**C**), and relative quantification (**D**). Immunostaining of MBP after exposure to soluble T3 (50 nM) (**E**), PLLA alone (**F**), PLLA + IBU + T3 (**G**), and relative quantification (**H**). Statistical analysis: one-way ANOVA and post-hoc test, ** *p* < 0.01, **** *p* < 0.0001. Abbreviations: CNPase, 2′,3′-cyclic-nucleotide 3′-phosphodiesterase; DIV, days in culture; Ibu, ibuprofen; iNOS, inducible nitric oxide synthase; MBP, myelin basic protein; PLLA, poly(l-lactic acid); T3, triiodothyronine; TNF-α, tumor necrosis factor-alfa.

The in vitro maturation of OPC was used to test the promyelinating properties of the drug-loaded scaffold. When exposed to inflammatory cytokines or LPS, OPC differentiation was blocked [14]. System maturation was induced by the culture medium conditioned by the PLLA + Ibu + T3 scaffold, as illustrated by the morphology (CNPase and MBP-positive cells, Figure 7A–C and Figure 7E–G, respectively) and counts of cells expressing mature oligodendrocytes markers (Figure 7D,H). The yield of maturation induced by the PLLA electrospun scaffold loaded with the two drugs is comparable to that of the positive control exposed to soluble T3.

**Figure 8:**
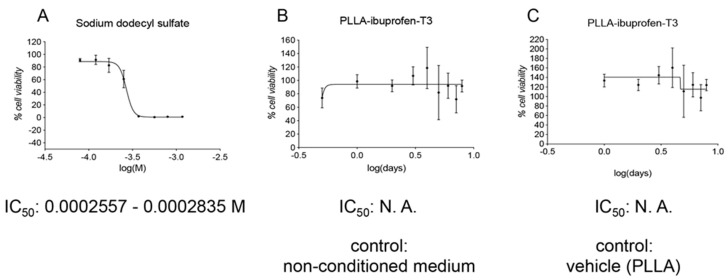
GLP toxicity in vitro evaluation of the delivery system. (**A**) The graph shows the cell viability normalized on the vehicle control group (100%) of cells exposed to different doses of sodium dodecyl sulfate. Concentrations on the x-axis are shown as the log of the Molarity; log(M). (**B**,**C**) The graphs show the cell viability normalized on the non-conditioned medium (100%; (**B**)) or the PLLA (vehicle)-conditioned medium (100%; (**C**)) of cells exposed to medium conditioned for different time lengths. Conditioning time on the x-axis is shown as the log of the conditioning days; log(days). For each graph, the IC_50_ value is shown as the Molarity, or as N. A. (non-applicable) if it was not possible to calculate the value.

Finally, we performed a toxicity evaluation of the complete pharmacological tool, composed of the PLLA electrospun scaffold and the two molecules. Culture media conditioned by the PLLA electrospun scaffold loaded with Ibu and T3 were used for the in vitro assay, using the standard medium or the PLLA electrospun scaffold without drugs as controls, treated as the test medium conditioned for the longest time (8 DIVs). As defined by the GLP guidelines, for each assay the sensitivity of the cell system to the positive control of cell death (SDS) has to be evaluated, and, also in this experiment, DITNC1 cells resulted in responsivity to cell death induction (Figure 8A). The exposure of the cells to media conditioned by PLLA electrospun scaffold loaded with Ibu and T3 did not reveal any toxic effect, both as compared to non-conditioned medium (Figure 8B) and PLLA vehicle (Figure 8C).

Notably, this study proved that the presence of T3 does not alter the IC_50_ of Ibu. Cells exposed to the electrospun scaffold never reached 0% of cell viability compared to the control vehicle groups. However, the exposure to the four wider tested surfaces produced a reduction in the MTT abs (almost 20% compared to the control vehicle group), concluding that the drug-loaded scaffold is not toxic for astrocytes.

## 4. Conclusions

In conclusion, a rational polypharmacology approach for the treatment of CNS acute injuries was exploited by incorporating two drugs, Ibu and T3, with complementary and potentially synergistic effects (anti-inflammatory and remyelinating) into a single polymeric scaffold. Based on previous in-house studies and an ad-hoc performed experiment, the effective concentrations of the two drugs, which at the same time show no toxicity, were preliminary determined. These were then loaded into the nanofibers, yielding scaffolds that were able to perform the intended sustained release. The cellular efficacy of the dual drug-loaded system restored OPC differentiation, as indicated by both CNPase and MBP-positive cells. Moreover, the anti-inflammatory efficacy, as evaluated in LPS-stimulated macrophages, is comparable to soluble drugs for TNF-α regulation, while it is superior for iNOS. To note, an improved functional recovery has been shown in an SCI rat model [13]. In our opinion, this workflow, by which preliminary biological studies provide the rational foundations and guide the development of the combinatorial treatment, could also be exploited for various other polypharmacological treatments of complex diseases. This would allow for addressing the critical need of developing polypharmacology in a more rational way, which is fundamental for a successful clinical translation.

## 5. Patents

An international patent application from the University of Bologna is pending (PCT/IT2018/000084).

## Figures and Tables

**Table 1:** Electrospinning parameters used to produce theoretical 5 wt% Ibu-loaded scaffolds, and drug encapsulation efficiency (EE).

Sample	Flow Rate (mL/h)	Needle-to-Collector Distance (cm)	Voltage (kV)	Ibu EE (% *w/w*)
PLGA 50:50	0.8	20	20	108.2 ± 0.8
PLGA 75:25	1.5	20	18	109.0 ± 3.2
PLGA 85:15	0.8	20	18	102.5 ± 2.1
PLLA-micro	2.4	20	18	101.0 ± 8.3
PLLA-nano	1.2	20	18	95.0 ± 1.6

## Supplementary Materials

The following are available online at https://www.mdpi.com/article/10.3390/pharmaceutics13060848/s1, Figure S1: chromatograms of Ibu A) Chromatogram of PLLA spiked with Ibu at the concentration of 50 ng/mL. B) Chromatogram of blank PLLA sample. Figure S2: chromatograms of T3 (A) Chromatogram of PBS spiked with T3 at the concentration of 20 ng/mL. (B) Chromatogram of blank PBS sample. Figure S3: (A) TGA analysis of PLLA (black), Ibu-loaded PLLA (blu), T3-loaded PLLA (red), Ibu (green), and T3 (pink). (B) DSC first heating scans of PLLA (black), Ibu-loaded PLLA (blue) and T3-loaded PLLA (red). (C) DSC first heating scans of Ibu-loaded PLLA before (black) and after (red) sterilization. (D) DSC first heating scans of T3-loaded PLLA before (black) and after (red) sterilization.
