# Supplementary material for: Design and In Vitro Study of a Dual Drug-Loaded Delivery System Produced by Electrospinning for the Treatment of Acute Injuries of the Central Nervous System
Source: Pharmaceutics. 2021 Jun 8;13(6):848. doi: 10.3390/pharmaceutics13060848 (PMC8227370; doi:10.3390/pharmaceutics13060848)
Supplement: Supplementary file 1 [file pharmaceutics-13-00848-s001.zip › pharmaceutics-1218103-supplementary.pdf]

pharmaceutics

Supplementary material: Design and In Vitro Study of a Dual Drug-Loaded Delivery System Produced by Electrospinning for the Treatment of Acute Injuries of the Central Nervous System

Luisa Stella Dolci, Rosaria Carmela Perone, Roberto Di Gesù, Mallesh Kurakula, Chiara Gualandi, Elisa Zironi, Teresa Gazzotti, Maria Teresa Tondo, Giampiero Pagliuca, Natalia Gostynska, Vito Antonio Baldassarro, Maura Cescatti, Luciana Giardino, Maria Letizia Focarete, Laura Calzà, Nadia Passerini and Maria Laura Bolognesi

1. HPLC-UV method for Ibuprofen analysis

Determination of Ibu content

Analysis of Ibu in PLGA (50:50 and 75:25) electrospun scaffolds

Samples were accurately weighted and solubilized in MeCN and diluted 1:1 with PBS (0.1 M pH 7.4). Then the samples were centrifuged for 10 minutes at 6000 r.p.m. and filtered through a syringe filter (diam. 25mm FLL/MLS PP membrane. NY 0.2μm; GVS Filter Technology Indianapolis, IN46268) and analyzed by HPLC-UV.

Analysis of Ibu in PLLA electrospun scaffolds

Samples were accurately weighted and solubilized in MeCN:DMSO (3:1 v/v) than added to MeCN:PBS (PBS 0.1M pH 7.4) in a ratio 1:4:5 v/v, heated up and sonicated for 15 minutes. Finally, the samples were centrifuged for 10 minutes at 10000 r.p.m., filtered through a syringe filter (NY 0.2 μm) and analyzed by HPLC-UV.

Analysis of Ibu from PBS buffer and DMEM cell culture medium

Samples were acidified with HCl conc than were centrifuged and filtered (0.4 μm NY filter); if necessary, the samples were diluted, then the drug content was analyzed by HPLC-UV.

Validation

The proposed method was validated according to the main requirements of the European guidelines [1] by evaluation of specificity, linearity, lower limit of quantification, accuracy and precision.

The specificity was confirmed by the absence of interfering compounds at the specific retention time of the analyte in the chromatograms obtained samples of the three matrices (PBS buffer, DMEM cell culture medium and PLLA). Figure S1 reports, as a representative example, the chromatograms obtained in PBS buffer.

Matrix-matched calibration curves (seven levels: 0, 0.05, 0.1, 0.5, 1, 5, 10, 20 μg/mL) were freshly prepared during different days to evaluate the linearity of the method. Peak areas were plotted against their concentrations applying a "no weighting" linear regression model, obtaining satisfactory coefficients of correlation (R2 >0.99).

The limit of detection calculated as LOD=3σ/s was 6.2 ng/mL and the limit of quantification calculated as LOQ=10σ/s was 20.8 ng/mL.

Accuracy and precision (intra- and inter-day) were tested by spiking blank matrix samples at three different concentrations (10, 1 and 0.1 μg/mL) in quintuplicate, and in three different days. The analysis of these QC samples in the three matrices proved the good accuracy (percentage difference always lower than ± 15%) and precision (intra- and inter-day CV% always below ± 15%) of the method. The recovery was tested at two different concentrations within the validation range (10 and 1 μg/mL), resulting always >98%.

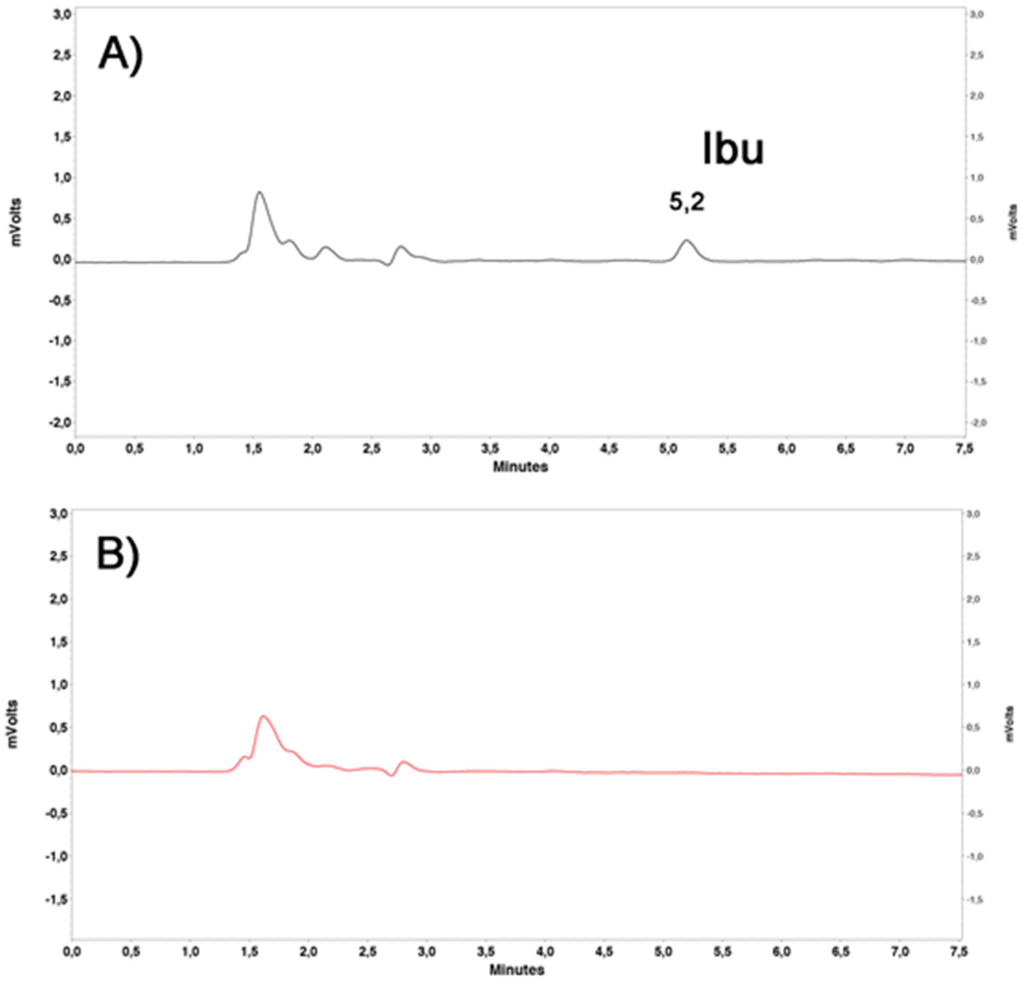

Figure S1. chromatograms of Ibu. (**A**) Chromatogram of PLLA spiked with Ibu at the concentration of 50 ng/mL; (**B**) Chromatogram of blank PLLA sample.

2. UPLC-MS/MS method for T3 analysis

Analysis of T3 from PBS buffer and DMEM cell culture medium

Sample clean-up was carried modifying a previously described procedure. Briefly, after conditioning the Oasis® HLB cartridge with 2 mL of MeOH and 2 mL of water, always avoiding the solid phase to go dry, 1 mL of sample was loaded. Once all the solution had passed through the column, the washing was performed with 3 mL of a mixture of water/MeOH (80/20, v/v), then vacuum was applied for 5 min to remove eventual residual drops. The analyte was eluted with 1 mL of MeOH containing 0.1% of formic acid in vial prior to analysis in UPLC–MS/MS.

Analysis of T3 from PLLA

3 mg of PLLA were extracted for three times with 1 mL of methanol and left in ultrasonic bath for 10 minutes. The three extract were pooled and evaporated under N2 stream to the volume of 1mL in volumetric flask prior to analysis in UPLC–MS/MS.

Validation

The proposed method was validated according to the main requirements the European guidelines [1] by evaluation of specificity, linearity, lower limit of quantification, accuracy and precision.

The specificity was confirmed by the absence of interfering compounds at the specific retention time of the analyte in the chromatograms obtained samples of the three matrices

(PBS buffer, DMEM cell culture medium and PLLA). Figure S2 reports, as a representative example, the chromatograms obtained in PBS buffer.

Matrix-matched calibration curves (seven levels: 0, 20, 50, 100, 200, 300, 500 ng/mL) were freshly prepared during different days to evaluate the linearity of the method. Peak areas were plotted against their concentrations applying a "no weighting" linear regression model, obtaining satisfactory coefficients of correlation (R2 >0.99).

The lower limit of quantification (LLOQ) of the method, corresponding to the lowest concentration with signal to noise (S/N) ratio >10, was 3 ng/mL.

Accuracy and precision (intra- and inter-day) were tested by spiking blank matrix samples at three different concentrations (25, 50 and 100 ng/mL) in quintuplicate, and in three different days. The analysis of these QC samples in the three matrices proved the good accuracy (mean concentration within 15% of the nominal values) and precision (intra- and inter-day CV% did not exceeded 15%) of the method.

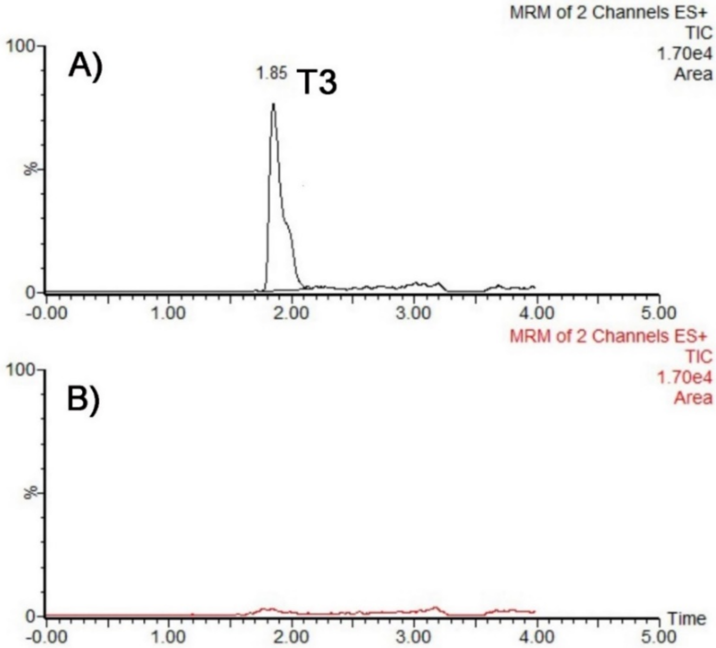

Figure S2. chromatograms of T3. (**A**) Chromatogram of PBS spiked with T3 at the concentration of 20 ng/mL; (**B**) Chromatogram of blank PBS sample.

3. Thermal characterization of drug-loaded nanofibers

To verify whether the physical properties of the nanofibers were affected by the inclusion of either Ibu or T3, thermal characterization was carried out on the Ibu-loaded and T3-loaded PLLA fibers and results were compared with those of pure PLLA fibers. It is specified that the thermal characterization was intentionally carried out on the single-loaded scaffolds rather than on the dual drug delivery system, to distinguish the possible effects of Ibu from those of T3 on the polymer properties. In addition, the thermal characterization was repeated after the sterilization with γ-rays.

Figure S3A reports the thermogravimetric analysis (TGA) of the drug-loaded fibers, of pure PLLA fibers and of the pure drugs. PLLA degrades in a single decomposition step, occurring in the range 300-400 °C. When loaded with either Ibu or T3, the degradation onset is slightly anticipated. Moreover, the presence of Ibu in the Ibu-loaded fibers can be detected by the appearance of a new weight loss of 4% in the range 100-200 °C, corresponding to the range of degradation of the pure Ibu and in line with the theoretical amount of 5 wt%. Differently, pure T3 degrades with a complex multi-step decomposition curve and its theoretical amount in the fibers (i.e. 0.6 wt%) is below the limit of detection of TGA analysis.

Figure S3B reports the first Differential Scanning Calorimetry (DSC) heating scan of both drug-loaded and unloaded PLLA. The presence of the drugs in the fibers slightly changes the thermal transitions of PLLA. The glass transition temperature (T_g) of PLLA, located at 64 °C in the pure fibers, decreased to 57 °C in the presence of 5 wt% of IBu and to 62 °C with 0.6 wt% of T3. Concomitantly, the temperature of cold crystallization (T_{cc}) decreased, from 83 °C of the pure PLLA, to 76 °C with the presence of Ibu, whereas it remains unchanged with the presence of T3. The melting temperature (T_m) slightly decreased in the presence of drugs (T_m of PLLA = 175°C, T_m Ibu-PLLA = 172, T_m T3-PLLA = 173°C). The plasticization effect of drugs well miscible with the polymers was previously reported [2,3], including the plasticization of PLLA by Ibu [4]. It is well-established that the extent of plasticization is dependent on the amount of plasticizer. In our study Ibu-loaded fibers are more extensively affect by the presence of the drug with respect to T3-PLLA loaded fibers as a consequence of the higher drug concentration used in the former. Overall, the change of thermal transitions is minimal and does not affect the applicability of the materials.

Figure S3C and S3D compare the first DSC heating scans of Ibu-loaded fibers and T3-loaded fibers, before and after the sterilization. It is evident that DSC traces of virgin samples are not modified by the γ-ray treatment, meaning that the polymer properties were maintained.

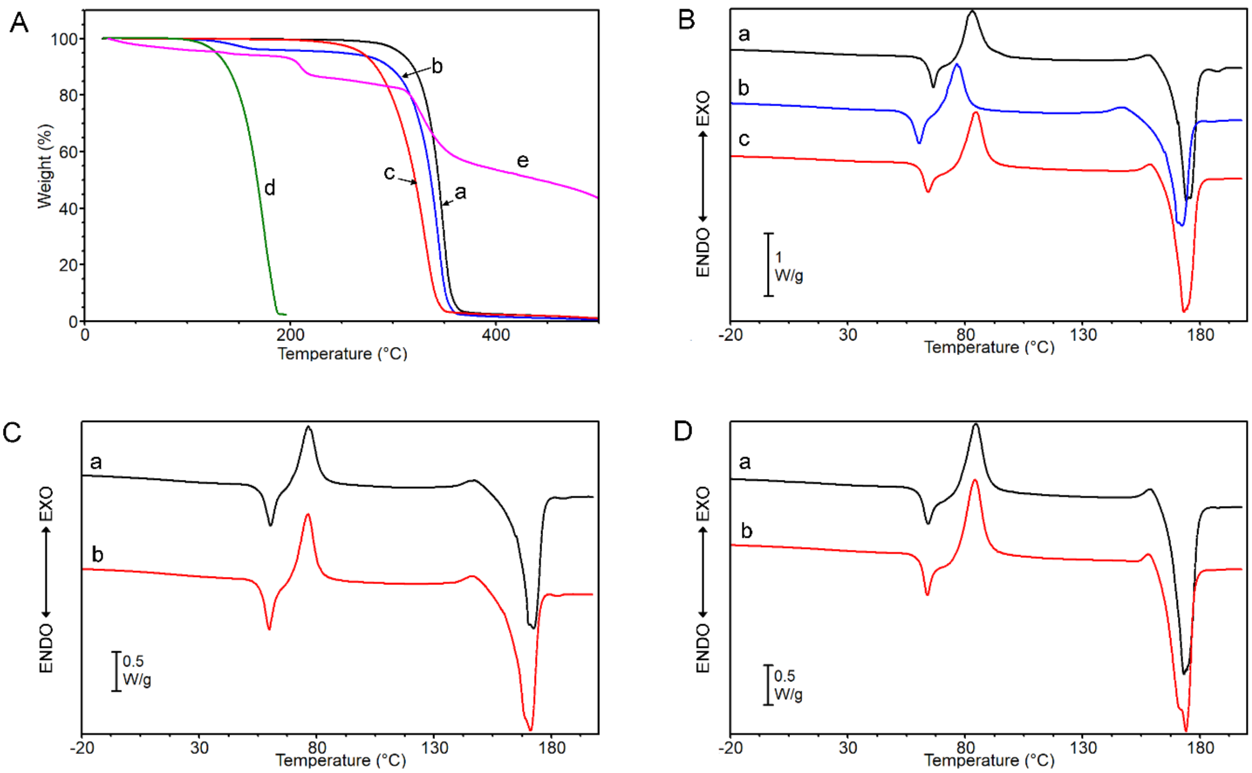

Figure S3. (**A**) TGA analysis of PLLA (black), Ibu-loaded PLLA (blu), T3-loaded PLLA (red), Ibu (green), and T3 (pink). (**B**) DSC first heating scans of PLLA (black), Ibu-loaded PLLA (blue) and T3-loaded PLLA (red). (**C**) DSC first heating scans of Ibu-loaded PLLA before (black) and after (red) sterilization. (**D**) DSC first heating scans of T3-loaded PLLA before (black) and after (red) sterilization.

References

1. European Medicines Agency. Draft Guideline Bioanalytical method validation. Available online: https://www.ema.europa.eu/en/documents/scientific-guideline/draft-guideline-validation-bionanalytical-methods_en.pdf (accessed on 17 March 2021).
2. Wu, C.; McGinity, J.W. Non-traditional plasticization of polymeric films. *Int. J. Pharm.* **1999**, *177*, 15-27.
3. Wu, C.; McGinity, J.W. Influence of Ibuprofen as a Solid-State Plasticizer in Eudragit® RS 30 D on the Physicochemical Properties of Coated Beads. *AAPS PharmSciTech* **2001**, *2*, 35-43.
4. Sun, C.; Zou, L.; Xu, Y.; Wang, Y. Ibuprofen-Loaded Poly(Lactic Acid) Electrospun Mats: The Morphology, Physicochemical Performance, and In Vitro Drug Release Behavior. *Macromol. Mater. Eng.* **2020**, *305*, 2000457.
